# The Antiseptic Properties of Tar Water

**Published:** 1878-09-20

**Authors:** Thos. R. Wright

**Affiliations:** Assistant Demonstrator of Anatomy in the Medical Department University of Georgia, at Augusta


### SOUTHERN MEDICAL RECORD.

VOL. VIII. ATLANTA, GA., SEPT. 20, 1878. NUMBER 9.

EDITORS:

THOMAS S. POWELL, M. D. W. T. GOLDSMITH, M. D. R. C. WORD, M. D.

*R. C. WORD, M. D., Managing Editor.*

SUBSCRIPTION—$2.00 PER ANNUM, IN ADVANCE.

☞ All Communications and Letters on Business connected with THE RECORD, for 1877 and 1878, must be addressed to the Managing Editor.

### *ORIGINAL AND SELECTED.*

### THE ANTISEPTIC PROPERTIES OF TAR-WATER, AS PROVEN BY THE FOLLOWING CASES IN THE PRACTICE OF PROF. DESAUSSURE FORD, M.D.

Reported by THOS. R. WRIGHT, M.D., Assistant Demonstrator of Anatomy in the Medical Department University of Georgia, at Augusta.

Tar-water having proven itself of such value as an antiseptic in the following cases of Dr. Ford, it occurred to me its value should be made known to the profession at large, but especially to my Southern brethren, who are able to obtain tar from the distillation of the wood of the yellow pine, its chief ingredient, at comparatively little or no expense. It can be prepared by any one in the following manner: Take a quart of *pine tar*, add to it a gallon of water and let it remain for forty-eight hours, when the water may be drawn off and used. The vessel can be refilled with water several times before that amount of tar will cease to impart its virtues. When made in large quantities, as at our City Hospital, five gallons of tar to the barrel of water, refilling the barrel as often as the water is taken out, until it ceases to have any tarry odor, or to be of a pale straw color. The antiseptic properties of tar-water are due to the hydro-carbons which abound in pine tar, but chiefly of these to pyroligneous acid and creasote, both of which seem to yield their well known antiseptic properties to water.

Tar-water is considered by Prof. L. A. Dugas to be one of the best antiseptics, and it was extensively used by him in the various hospitals under his care during the late civil war. Then it was, he saw its value. Quoting from the Doctor, who says: "The efficacy of tar-water in treating hospital gangrene, is perfectly marvellous. Not only does it stop the mortification, sloughing and horrible odor, but relieves the awful pain caused by the gangrenous process attacking sound tissues."

It was used by him in the following way: After cutting away all dead tissue the wound was thoroughly mopped or washed out with tar-water, and cloths saturated with it were kept constantly on the wound.

From the time this treatment was inaugurated by Prof. Dugas, there was not a single death from gangrene in his wards, and it was astonishing how rapidly granulations began to form and the wounds to assume a healthy aspect. The wards, which were intolerably offensive, from the number of cases of gangrene in them, also became completely deodorized as soon as the tar-water treatment was begun.

This article has long been known.* The Bishop of Cloyne, writing in 1747, extals it very highly in many diseases, especially fevers. And Dr. Cullen, writing about the same time also speaks of its value. Yet, it is to Prof. Dugas that we are indebted for its discovery as an antiseptic and disinfectant, and for its value in treating hospital gangrene.

Tar-water is considered by Dr. Ford and m: self, equal in its antiseptic and deodorizing properties to carbolic acid, besides being a great deal cheaper; an item of no little importance at the present time—feeling perfectly satisfied with the results obtained from using it, as will be indicated. We have never tried it according to the Lister method of antiseptic surgery, but feel confident if it were so tried, it would be as successful in an equal number of cases. It is used in dresssing all surgical cases in the City and Freedman's hospetals of this city, just as the carbolized solutions are in the surgical wards of the large hospitals of the North, and with about as good results.

The following cases are but a few wherein its virtue has been proven:

* Rees' Cyclopedia, Vol. xxxvi.

(This case will be reported in full by Prof. Ford in the American Journal of Medical Sciences).

CASE 1. Martha Callahan. From this patient Dr. Ford removed a teratoma or teratora tumor from the side of the face and neck. After removing the tumor and bringing the flaps together, a sac necessarily remained; a tent was placed in the most dependent portion of the wound for drainage, and that the sac could be daily irrigated with tar-water, which was regularly done, and there being little or no suppuration, the tent was accidentally left out for a day and the irrigation omitted, the wound closed, and at our next visit the sac was filled with pus, which was let out by incision, and was horribly offensive, until some tar-water was thrown into the vessel containing it, when it was immediately deodorized. After this the irrigation was regularly kept up, the amount of suppuration being almost nothing from the sac.

CASE 2. Thos. G———, white. Compound Potts fracture of left leg. Dr. Ford was called to see this case in consultation by his attending physician, about one month after the injury was received. The wound was found in such a condition that after consultation, it was decided to amputate the limb, as the only means of saving the patient's life. Preparation was immediately made for operating, and wishing to give tar-water another trial, a supply of it was obtained and used as will appear.

Upon close examination, it was found that pus had burrowed up the limb as high as its middle third, where it was decided to amputate. Our assistant was directed to keep a constant current of tar-water pouring over the limb from a large sponge, during the entire operation, which was faithfully done. As the knife passed through the skin, the pus spouting out from beneath it, and the fascia was very offensive until a sponge full of tar-water deodorized it, as if by magic. So completely and quickly was this done, that it was immediately remarked by those present. The operation was completed, vessels ligated, and flaps brought together by four silk sutures and adhesive plasters. The irrigation with tar-water being kept up continuously. The stump was then dressed with compresses wet with tar-water and bandaged up.

The patient reacted well, and was left with directions to keep the dressing wet with tar-water. The case was then turned over to me to dress. Upon visiting him on the third day after the operation, there was not the least odor about the dressings, and upon removing them there was comparatively no suppuration, possibly a teaspoonful of bloody ooze, mixed with a little pus being found in the compresses, the pus here, I believe to have come from the abscess through which the knife passed, for at no subsequent dressing was there any pus found. The stump looked beautifully. After irrigating well with tar-water, it was dressed as at first, the same method of dressing being kept up every third day for two weeks, after which time, the wound being in such a fine condition, the dressing of it was left to his father. At the present writing, five weeks after the operation, he is going about on crutches with his stump cicatrized over.

Case 3. Ellen McD——, factory operative. While attending to her loom her right forefinger was caught in the gearing and cut and mangled up as far as the second joint, the ungual phalanx being ground up. Dr. Ford, upon seeing the case, and wishing to save the finger if possible, brought the parts together as well as could be done, with four fine iron wire sutures, and dressed the wound with compresses saturated in tar-water, telling her to keep them so. I also dressed her finger for two weeks, at the end of which she was able to take care of it herself.

In this, as in the other cases mentioned, there was, at no time, any odor or appreciable suppuration. At the present time, she too, is entirely well.

The next case is one which was seen by Prof. L. D. Ford and myself.

Case 4. Hester ——, a colored woman, cook. While attending to her duties in the house of her employer, tripped and fell one evening, while carrying a burning kerosene lamp, which was broken, the oil igniting, and her clothes taking fire—burning both arms, her breast and back very badly. Immediately upon seeing the case, she was punctured with half a grain of morphine, and cloths saturated in spirits of turpentine, were applied to the burned surfaces. (The application of turpentine to burns is a treatment introduced by the Dr., and a very valuable one it is.) Upon removing these cloths the patient's arms and body were enveloped in large compresses, which were ordered to be kept wet with a solution of chloride of soda —it being at night, and in a private family, the soda was more easily obtained than tar-water. The next day she was removed to the freedman's hospital, when the soda solution was superseded by tar-water, the dressings being kept wet with it.

Upon dressing the case three days after the accident, there was no odor and very little suppuration. The wounds were washed and dressed as before, and the cloths kept wet. On the sixth day, we dressed her again. There was still no odor about the dressings, although upon removing them we found that ulceration had begun to take place, and there was some suppuration. She was again dressed, but succumbed to pneumonia on the ninth day.

This case, from the extent of the injury, was considered one of the best wherein the antiseptic properties of this article were illustrated. I would state, that all these cases, save the first, were treated during very hot weather.

Vastly more could be written on this subject, but if I am able to call the attention of the profession to the value of tar-water, as an antiseptic and disinfectant, I am content.

———

Oxide of zinc is recommended as a specific for the tremor of chronic alcoholism.

